# Beyond Blast Injury: Occupational Hygiene, Safety, and Toxicology Considerations for Mixed-Metal and Energetic-Chemical Exposures to Explosive Ordnance Disposal Personnel

**DOI:** 10.3390/toxics14050379

**Published:** 2026-04-28

**Authors:** Bryan G. Fry, Kelly Johnstone, Stacey Pizzino

**Affiliations:** 1 Adaptive Biotoxicology Laboratory, School of the Environment, University of Queensland, St Lucia, QLD 4072, Australia; 2 Occupational Hygiene & Exposure Science Laboratory, School of the Environment, University of Queensland, St Lucia, QLD 4072, Australia; k.johnstone2@uq.edu.au; 3 School of Public Health, University of Queensland, Brisbane, QLD 4006, Australia; s.pizzino@uq.edu.au

**Keywords:** explosive ordnance disposal, explosive remnants of war, unexploded ordnance, metal, carcinogen, occupational hygiene

## Abstract

Explosive ordnance (EO), including AXO (abandoned explosive ordnance), IEDs (improvised explosives devices), and UXO (unexploded ordnance), are widely recognised for their blast and fragmentation hazards, but they also represent a persistent and under-addressed source of occupational chemical exposure for explosive ordnance disposal (EOD) personnel. EOD core activities liberate mixed metals and energetic chemicals, resulting in exposures that are multi-route (inhalation of dusts and fumes, dermal loading amplified by sweat and glove occlusion, and ingestion via hand-to-mouth transfer during eating, drinking, or smoking) and multi-temporal (repeated low-dose background plus task-driven spikes), as well as chemically complex. Clinically, this can present as syndromic overlap across acute and chronic domains, with symptoms that are easily misattributed to heat stress, dehydration, infection, or fatigue. Acute effects of concern include neurotoxic presentations (headache, dizziness, confusion, tremor, and seizure), respiratory and mucosal irritation following dust or fume events, gastrointestinal symptoms, and patterns suggestive of acute hepatic or renal stress, particularly when high-intensity tasks occur in hot environments that compound physiologic strain. Chronic outcomes relevant to repeatedly exposed EOD personnel include renal function decline, neurocognitive effects that can degrade operational decision making and safety, persistent haematologic abnormalities, and endocrine disruption signals, with long-latency risks requiring cautious interpretation given sparse longitudinal data and confounding co-exposures. This review synthesises the current evidence base through an EOD lens and translates it into pragmatic clinical and programmatic actions: task-based exposure characterisation; tiered biomonitoring and medical surveillance aligned to operational tempo; incident-triggered assessment pathways after high-residue events; and prevention strategies that work under field constraints, including contamination control zones, hygiene enforcement, glove and respiratory protection optimisation, tool and vehicle decontamination, and measures to prevent secondary transfer and take-home exposure. The central takeaway is practical: EOD programs can reduce morbidity and improve readiness by treating explosive ordnance as a chemical mixture exposure problem, adopting mixture-aware clinical triage, and embedding surveillance and controls that match how EOD work is actually performed.

## 1. Introduction

Explosive ordnance, including abandoned explosive ordnance (AXO), unexploded ordnance (UXO), and improvised explosives devices (IEDs), are usually discussed as kinetic hazards [1]. However, they also constitute a persistent, preventable chemical source of occupational exposure for explosive ordnance disposal (EOD) personnel. Render-safe procedures (including cutting/drilling) and demolition EOD work repeatedly places operators in high-intensity, direct contact with munitions, corroded casings, chemical leakage, disturbed soils, and post-detonation debris. These settings contain mixtures of metals and metalloids derived from alloys, primers, fuses, and corrosion products, as well as energetic chemicals and transformation products from fills and propellants [2,3,4,5,6,7,8,9,10,11,12,13,14,15,16,17,18,19,20,21,22]. Complementing this, the site-specific geochemistry and disturbance regimes shape the fate and transport of the mixed metals and energetic chemicals [23].

The purpose of this review is to reframe explosive ordnance as an EOD occupational hazard in which complex mixture exposure is the default condition, not an exception. Traditional “single chemical” thinking is a poor fit because EOD exposures are multi-route, multi-temporal, and mechanistically convergent. In the field, inhalation occurs via dust during excavation and brushing and via fumes and ultrafines during hot work and post-detonation inspection. Dermal loading is amplified by sweat and glove occlusion, with contamination transfer during doffing and handling of tools, vehicles, and personal items. Ingestion occurs through hand-to-mouth transfer during eating, drinking, smoking, or face wiping. These routes combine across repeated deployments, producing a cumulative dose with intermittent high-exposure events, leading to additive or more complex outcomes resulting in disease endpoints that often cannot be mapped cleanly to one agent [24,25,26,27,28,29,30,31,32,33,34,35,36,37,38]. For EOD programs, the practical implication is that hazard identification, clinical reasoning, and surveillance design must be mixture aware and task linked.

This review therefore defines its scope around EOD personnel exposed to mixed metals plus energetic chemicals, and it focuses on clinically relevant acute and chronic outcomes. Acute presentations of particular operational concern include neurological syndromes; eye, respiratory, and mucosal irritation after dust or fume events; gastrointestinal symptoms; and patterns consistent with hepatic or renal stress. Neurotoxicity is a potential pathophysiological outcome in scenarios involving mishandling, accidental ingestion, or extreme contamination events. For example, generalised seizures have been documented after exposure to RDX (Research Department Explosive)-containing plastic explosive [39,40,41,42,43,44]. For TNT (2,4,6-Trinitrotoluene), occupationally exposed worker studies describe systemic complications including toxic hepatitis, haematologic effects, and ocular findings, underscoring that energetic chemicals can have clinically meaningful endpoints beyond irritation [45,46,47,48,49]. Chronic outcomes relevant to repeatedly exposed EOD personnel include cardiovascular issues, renal function decline, and neurocognitive effects that can degrade decision quality and safety. For example, cumulative exposure to lead has been linked to nephrotoxicity, reinforcing the need for longitudinal kidney surveillance when EOD personnel are chronically exposed [50,51,52,53,54,55].

A key operational point is that EOD personnel symptom patterns from explosive ordnance acutely or chronically exposed to mixed metals or energetic chemicals, can overlap with heat illness, dehydration, and infection. This is especially true in tropical or austere settings, where physiologic strain and infectious or chronic disease burdens can confound the interpretation of non-specific symptoms and laboratory signals. As such, the final aim of the review is translational: to show how surveillance and prevention can be operationalised in EOD contexts through task-based exposure characterisation, tiered biomonitoring and medical surveillance aligned to operational tempo, and field-feasible controls focused on contamination management, hygiene enforcement, and PPE strategies that work under heat and dexterity constraints. It is important to note that there are differences in PPE practices globally, not only in a developed world versus a developing world context, but also in terms of task-specific PPE. For example, PPE for landmine clearance is typically very different than for chemical weapons. There are also implications in settings where the corrosion of devices may make it difficult to determine the device and therefore the risk profile.

This review has four aims: (1) to reframe EO as an occupational chemical mixture hazard rather than a single-agent risk; (2) to synthesis evidence on the operational exposure pathways encountered in EOD work; (3) to characterise the acute and chronic health effects arising from exposures; and (4) to translate this evidence into pragmatic control strategies that reflect how EOD tasks are performed in diverse operational settings.

This article is a narrative review designed to synthesise evidence relevant to occupational hygiene, safety, and toxicology in explosive ordnance disposal (EOD) contexts. The literature was identified through targeted searches of major bibliographic databases, including PubMed, Scopus, and Web of Science, supplemented by Google Scholar and backward and forward citation tracking of key papers and authoritative reports. Search terms combined concepts related to explosive ordnance disposal, unexploded ordnance, abandoned explosive ordnance, improvised explosive devices, metals, energetic chemicals, dust, fumes, biomonitoring, surveillance, and acute and chronic health effects. Priority was given to peer-reviewed human studies, systematic reviews, and authoritative toxicological and occupational health sources. Because EOD-specific studies remain limited, relevant evidence from adjacent occupational and operational settings, including ammunition manufacture, demilitarisation, welding, firing ranges, and contaminated site work, was also included where it informed plausible exposure pathways, toxicological interpretation, or surveillance design. Studies were selected on the basis of relevance to EOD tasks, exposure routes, health outcomes, and prevention or monitoring implications. This review was not conducted as a formal systematic review and did not use PRISMA-based screening or meta-analytic methods.

## 2. Operational Exposure Context in Explosive Ordnance Disposal Work

EOD operations create a distinctive operational exposure context in which contact with contaminated media is both task driven and recurrent. During excavation and soil disturbance, ordnance is physically mobilised along with surrounding soils and fine particulates, producing inhalable and respirable fractions relevant to worker exposure [13,21,23,56,57,58,59,60,61,62]. Excavation by hand tools or brushing of corroded surfaces can therefore generate short-duration but high-intensity dust clouds containing mixed metals and residual energetic chemicals. Because EOD operators often work in close proximity to the source, breathing zone concentrations during these micro-tasks may exceed background environmental levels documented in static monitoring studies.

Surface brushing and cleaning further increase exposure potential by dislodging corrosion products and crystalline residues that have formed on casings, fuses, and surrounding soil aggregates. Energetic chemicals can adsorb to particles yet remain bioaccessible, particularly when soil is disturbed or wetted and dried repeatedly [63,64,65,66]. These characteristics are operationally relevant because brushing and manual manipulation create conditions conducive to inhalation and hand contamination (including potential dermal absorption issues), establishing parallel inhalation and ingestion pathways.

Render-safe procedures and hot work introduce an additional exposure domain. Cutting, drilling, plasma cutting, or gouging of corroded metal can generate metal fume and fine to ultrafine particles, formed when vaporised metal condenses in air, leading to breathing zone exposures [67,68,69,70]. In parallel, energetic chemicals present in casings, fuses, and corrosion matrices can be thermally degraded or frictionally decomposed during high-energy cutting or drilling, yielding complex suites of gaseous and condensed-phase products, including species with clear toxicologic relevance such as nitrogen oxides and nitrile- or aldehyde-class products in nitramine decomposition studies [71,72,73]. While controlled demolition consumes much of the energetic mass, it does not guarantee chemical “elimination” at the site. For example, field detonation studies demonstrated measurable RDX and TNT residues deposited after both live-fire and blow-in-place protocols, with residue loads strongly influenced by detonation efficiency and scenario [74]. Residues can persist as particles and chunks, particularly after low-order or disrupted events, and these particles expand the contamination footprint through subsequent physical weathering and redistribution [75,76]. This matters operationally because EOD personnel remain in proximity during staging, detonation setup, and post-blast inspection, when dust resuspension and fragment handling can reintroduce inhalation and hand contamination opportunities. Post-detonation and post-disturbance soils can contain both parent compounds and transformation products [77,78,79,80,81].

Vehicle transport and staging contamination represent an often overlooked but critical pathway. Tools, gloves, and ordnance fragments handled in contaminated zones can transfer residues into vehicles and staging areas, creating secondary exposure environments, consistent with the broader “take-home” contamination literature where workplace dust is carried on clothing and equipment and subsequently contaminates vehicles and other nominally clean spaces [82,83,84]. For example, research on metal transport from firing ranges demonstrates that particulate contamination can persist on surfaces and in dust reservoirs, with repeated resuspension and ongoing exposure potential unless stringent housekeeping and contamination controls are applied [84,85]. This creates conditions for chronic low-dose exposure via dermal–oral routes, including hand-to-mouth transfer, which can be a major contributor to systemic uptake [86,87]. Repeated loading and unloading of equipment may similarly seed enclosed cabins with fine particulates that can later be inhaled when disturbed and can contribute to ingestion risk through hand contamination [83,88].

Environmental modifiers substantially influence the magnitude and biological relevance of these exposures to EOD work practices. Heat and humidity increase cutaneous vasodilation and skin blood flow and drive sweating, changing the skin microenvironment and contact conditions for contaminants [89,90,91]. Increased skin temperature and hydration can also increase percutaneous absorption for some chemicals [92,93,94]. Glove occlusion, while protective against gross contamination, creates a microenvironment of elevated humidity and temperature that increases stratum corneum hydration, alters barrier function, and can enhance penetration or transfer of soluble contaminants to the stratum corneum [95,96,97,98]. Repeated donning and doffing increases the likelihood of hand and wrist contamination (including contamination of inner glove surfaces through transfer during removal), and this creates a pathway to secondary ingestion through subsequent hand-to-mouth contact during eating, drinking, or face wiping [99,100,101,102]. Oral uptake relevance is further supported by occupational evidence that hand and mouth contamination contributes materially to systemic absorption [86] and by experimental estimates of hand-to-mouth transfer efficiency for particulate metals [103].

In practical terms, EOD exposure is not static. It is shaped by task type, weather, and site history, which aligns with the core exposure science principle that exposure characterisation must reflect real-world contact patterns rather than static concentrations alone. Hydrological and geochemical factors significantly modify exposure risk. Flooding and intense rainfall can rework contaminated surface soils and fine particles, changing both mobility and persistence of munitions constituents. This enables migration from soils into groundwater, with transport and attenuation controlled by site hydrology, soil properties, and subsurface conditions [65,104,105]. Fate depends on environmental conditions, including redox potential and pH, which are directly relevant to flood-driven oxygenation and subsequent reducing phases in wet soils and shallow aquifers [106,107,108]. Soil pH, organic matter, and mineralogy also affect adsorption–desorption equilibria and bioaccessibility for both metals and energetic chemicals [109,110,111,112,113,114].

Direct task-resolved quantitative exposure data for EOD personnel remain sparse, so precise EOD-specific exposure ranges cannot yet be stated with confidence. However, the exposure science literature from analogous settings supports a comparative interpretation of likely relative intensity across EOD tasks. Dry excavation, brushing of corroded surfaces, hot work on contaminated metal, and immediate post-detonation inspection are likely to represent the highest short-term inhalation intensity because they generate mechanically resuspended dust, metal-rich fumes, or ultrafine particles in close proximity to the breathing zone. By contrast, vehicle, staging area, and tool contamination are more plausibly lower-intensity but recurrent secondary exposure scenarios that sustain dermal and ingestion pathways over longer timeframes. Comparison with single-agent occupational exposure limits is therefore informative only in a limited sense at present because EOD exposures are mixed, episodic, task conditional, and not yet characterised by sufficient personal monitoring data.

## 3. Chemical Composition of Explosive Ordnance Residues Relevant to Explosive Ordnance Disposal

### 3.1. Mixed Metals

Explosive ordnance residues present EOD personnel with a mixed-metal exposure profile that reflects the full explosive train and hardware, with metals occurring across casings, projectiles, cartridge components, and pyrotechnic and primer compositions. Key contributors include steel and copper–zinc jackets and penetrators, as well as lead alloys commonly hardened with antimony, with co-contaminant metals in impacted soils frequently including antimony, copper, arsenic, zinc, and nickel [115,116]. Primer and initiator chemistries can add lead, barium, and antimony-bearing particulates [117,118]. Field weathering matters because fresh fragments expose reactive metal surfaces that oxidise and form secondary mineral phases, changing speciation and increasing the fraction that partitions to soil fines or mobilises into porewater and runoff, with mobilisation modulated by soil matrix, redox, and saturation state [115,116]. In parallel, primer residues can generate very fine particles through high-temperature vaporisation and condensation, producing spherical metal-bearing particulates that include the classic Pb-Ba-Sb signature and can extend into the submicron range [119].

### 3.2. Energetic Chemicals and By-Products

Energetic chemicals also span multiple classes with distinct fate and effect profiles. Nitroaromatics (notably TNT and related dinitrotoluenes and aminodinitrotoluenes) are important because they undergo metabolic activation and redox cycling that generates reactive intermediates and reactive oxygen species, creating a bridge between exposure and oxidative damage endpoints [45,120,121]. Nitramines such as RDX and HMX are seizure-inducing neurotoxins through both oral and inhalation exposure [122,123,124,125,126]. Oxidisers such as perchlorate have endocrine-disrupting effects, with heightened concern for pregnancy and early development contexts [127,128,129]. Finally, combustion and detonation by-products can generate complex aerosols and soot-associated organics, including PAHs and metal-bearing particulate matter, which become relevant during open burning or incomplete combustion scenarios [130,131,132]. Co-exposure is relevant. For example, TNT and RDX together can produce interacting toxicity patterns in vivo, reinforcing the need to treat energetics as mixture hazards rather than isolated analytes [133].

### 3.3. Convergent Toxicity

For risk characterisation, the default assumption is typically concentration (dose) addition for similarly acting stressors and independent action for dissimilarly acting chemicals because these two concepts are the backbone for predicting joint toxicity from component data [134,135,136]. As such, it is a pragmatic approach to treat synergy as a risk, especially when operational triggers increase co-exposure intensity or when endpoints converge [137]. Mechanistically, mixed metals and energetic chemicals converge on oxidative stress, mitochondrial dysfunction, inflammatory signalling, and immunotoxic pathways, manifesting as overlapping renal, neurologic, and haematopoietic findings rather than a single clean clinical picture [51,138,139,140,141,142,143,144,145,146,147,148,149]. Clinically, this supports surveillance focused on clusters rather than single endpoints because mixtures are more likely to produce multi-system patterns than clean single-agent signatures. That is consistent with the broader environmental health mixtures framing that emphasises cross-domain outcomes and diagnostic ambiguity under co-exposure [25]. It also aligns with exposure science guidance that exposure characterisation and control strategies must match real-world activity patterns rather than static single-chemical assumptions [111].

### 3.4. Toxicological Profiles of Metals Found in Explosive Ordnance

The risk matrix for metal exposure is influenced by a range of factors including acute versus chronic exposure, relative bioavailability (which may vary due to the matrix the metal is embedded into), the chemical speciation of the metal, and the degree of mixed-metal composition, as some metals are not highly toxic but are sensitisers.

#### 3.4.1. Aluminium (Al)

Acute exposure via dust inhalation may cause eye, nose, and throat irritation, cough, and short-term bronchitic symptoms, which can occur with high particulate loading [150]. Chronic exposure via dust inhalation has been linked to pulmonary aluminosis, including interstitial lung disease patterns reported in occupational cohorts [150,151]. Acute exposure via hand-to-mouth transfer results in oral toxicity, usually gastrointestinal effects only at higher intakes, consistent with the generally low absorption for many aluminium forms [150,152]. Chronic exposure via hand-to-mouth transfer leading to systemic effects (renal impairment, bone disease and neurologic toxicity) is most clearly documented in high-exposure settings [150,152].

#### 3.4.2. Arsenic (As)

Acute exposure via dust inhalation may lead to respiratory irritation, including severe bronchitis-like symptoms in higher exposures [153]. There is strong evidence for chronic exposure via dust inhalation leading to increased respiratory cancer risk [153,154]. Acute exposure via hand-to-mouth transfer may result in gastrointestinal toxicity, with symptoms including nausea, vomiting, diarrhoea, and abdominal pain, and can also cause cardiovascular instability and neurologic signs [153]. Chronic exposure via hand-to-mouth transfer may lead to multi-system toxicity, including dermatologic effects, peripheral neuropathy, cardiovascular disease, nephrotoxicity, and multiple cancers [153,155,156].

#### 3.4.3. Antimony (Sb)

Acute exposure via dust inhalation may lead to irritant effects on upper airways, while chronic exposure via dust inhalation has been associated with pneumoconiosis-like findings and chronic bronchitic symptoms in occupational histories [157]. Trivalent antimony is classified by the International Agency for Research on Cancer as a Group 2A carcinogen, with inhalation leading to lung cancer [157,158]. Acute exposure via hand-to-mouth transfer at higher oral doses can cause gastrointestinal upset, and severe poisonings can include cardiac and systemic effects, with relevance depending on compound solubility and dose [157]. Chronic exposure via hand-to-mouth transfer leading to chronic oral toxicity is less well characterised than inhalation outcomes, but compound-specific long-term exposure effects include liver and cardiovascular endpoints [157].

#### 3.4.4. Barium (Ba)

Acute exposure via dust inhalation may have irritant effects on eyes and airways, with systemic toxicity depending on compound solubility [159]. Chronic exposure via dust inhalation is less well defined for insoluble barium particulates, with risk interpretation strongly compound specific [160]. Acute exposure via hand-to-mouth transfer of soluble barium salts is the dominant acute hazard scenario, causing profound hypokalaemia, weakness or paralysis, and malignant arrhythmias [160,161]. Chronic exposure via hand-to-mouth transfer outcomes are less consistently characterised than acute poisoning, with compound solubility and repeated dose driving relevance [160].

#### 3.4.5. Cadmium (Cd)

Acute exposure via dust or fume inhalation may lead to chemical pneumonitis and pulmonary oedema, with the risk level rising if tasks generate fresh fumes or fine aerosols, while chronic inhalation exposure can lead to lung injury [162,163]. Acute exposure via hand-to-mouth transfer typically results in gastrointestinal irritation [164]. Chronic exposure via hand-to-mouth transfer often leads to kidney tubular injury as a core endpoint; bone demineralisation and fracture risk often follow, partly via renal mechanisms, and it is also a cardiovascular injury risk factor [164,165]. Cadmium is also a human carcinogen in occupational settings [164,166].

#### 3.4.6. Chromium (Cr)

Total soil chromium does not indicate speciation, so occupational interpretation benefits from follow-up speciation where feasible to determine the relative presence of the toxic hexavalent chromium form Cr(VI) [167,168]. Acute exposure via dust inhalation of hexavalent chromium may have acute outcomes, including airway irritation, rhinitis, asthma, and nasal ulceration [167]. Chronic exposure via dust inhalation of hexavalent chromium can be carcinogenic, with lung cancer and sinonasal cancers being the main outcomes [167,169]. Acute exposure via hand-to-mouth transfer may result in gastrointestinal irritation and systemic toxicity; this is less likely from incidental soil ingestion unless concentrations and bioaccessibility are high [168]. Chronic exposure via hand-to-mouth transfer leading to oral carcinogenicity is less likely than inhalation carcinogenicity for Cr(VI). However, dermatitis from chronic contact is relevant in contaminated soil environments [167,168].

#### 3.4.7. Copper (Cu)

Acute exposure via dust inhalation may lead to irritant respiratory effects [170]. Chronic exposure via dust inhalation can maintain airway inflammation [170]. Acute exposure via hand-to-mouth transfer typically causes gastrointestinal upset, which at higher doses may lead to nausea, vomiting, diarrhoea, and abdominal pain [171]. Chronic exposure via hand-to-mouth transfer can contribute to hepatic and systemic toxicity, but this is more relevant to sustained higher intakes than to low incidental soil ingestion [172].

#### 3.4.8. Iron (Fe)

Acute exposure via dust inhalation may lead to “nuisance dust” type of irritation at high dust load [173]. Chronic exposure via dust inhalation can be associated with respiratory symptoms and mixed-dust effects, causing pulmonary siderosis in severe cases [173]. Acute exposure via hand-to-mouth transfer leading to clinically important acute iron poisoning generally requires substantial ingestion, with incidental soil ingestion is unlikely to reach that range in adults. Chronic exposure via hand-to-mouth transfer typically does not lead to systemic iron overload. For EOD, iron is mainly a marker of particulate exposure and lung deposition rather than ingestion toxicity [173].

#### 3.4.9. Lead (Pb)

Acute exposure via dust inhalation can lead to systemic toxicity, with symptoms including headache, abdominal pain, cognitive changes, and haematologic effects from disrupted haem synthesis [174]. Chronic exposure via dust inhalation is associated with hypertension and cardiovascular disease, kidney disease, neurocognitive effects, and reproductive effects [50,51,52,53,54,55,175,176,177]. Acute exposure via hand-to-mouth transfer is a major pathway in dusty worksites and may lead to “lead colic”-type abdominal pain and neurologic features [174]. Chronic exposure via hand-to-mouth transfer has been linked to increased cardiovascular mortality [175,176]. Carcinogenicity note: Inorganic lead compounds are classified by the International Agency for Research on Cancer as Group 2A carcinogens [178,179].

#### 3.4.10. Manganese (Mn)

Acute exposure via dust inhalation at higher doses may lead to airway irritation and bronchitic symptoms [180]. Chronic exposure via dust inhalation has neurotoxicity as the signature chronic outcome; prolonged inhalation can produce manganism, a parkinsonism phenotype with motor and neurobehavioural impairment [180,181]. Acute exposure via hand-to-mouth transfer has acute systemic toxicity from incidental ingestion, which is less typical than inhalation-driven effects [181]. Chronic exposure via hand-to-mouth transfer can contribute to neurologic risk in some contexts, but inhalation remains the critical occupational pathway [181].

#### 3.4.11. Mercury (Hg)

Acute exposure via inhalation as elemental mercury vapour is the highest inhalation hazard, with acute toxicity leading to respiratory injury and neurologic symptoms [182]. Chronic exposure via inhalation of vapour is associated with tremor and neurobehavioural deficits [182,183]. Acute hand-to-mouth risk depends on speciation: inorganic mercury salts can be corrosive and nephrotoxic at high doses, soil-bound mercury is usually inorganic and less bioavailable, while organic mercury (methylmercury) is more readily absorbed and neurotoxic [183]. Chronic exposure via hand-to-mouth transfer has differential effects depending on mercury species; in occupational settings, inhalation is usually more important than low-level incidental ingestion unless contamination is extreme or bioavailable [182].

#### 3.4.12. Nickel (Ni)

In addition to its own toxic effects, nickel acts as a respiratory sensitiser, thereby potentiating the toxic effects of other metals. Acute exposure via dust inhalation can lead to occupational asthma [184]. Chronic high exposure via dust inhalation of certain nickel compounds is associated with lung and nasal cancers [185,186]. Acute exposure via hand-to-mouth transfer can contribute to systemic exposure, and acute skin exposure in sensitised individuals may trigger dermatitis [187]. Chronic exposure via hand-to-mouth transfer can perpetuate allergic contact dermatitis in sensitised workers, and this often coexists with other atopic disease [187]. Carcinogenicity note: Nickel compounds have been classified by the International Agency for Research on Cancer as Group 1 carcinogens [179].

#### 3.4.13. Selenium (Se)

Acute exposure via dust inhalation of high particulate loads may lead to irritation of the eyes and upper airways, with additional symptoms including bronchitic symptoms [188,189]. Chronic exposure via dust inhalation can lead to persistent airway irritation and chronic bronchitis-type symptoms, and systemic features can occur if absorbed doses are high enough [188]. Acute exposure via hand-to-mouth transfer at higher doses typically causes gastrointestinal toxicity, with nausea, vomiting, abdominal pain, and diarrhoea as the common early features, and more severe poisonings can include systemic effects [188]. Chronic exposure via hand-to-mouth transfer may lead to selenosis, characterised by hair loss, brittle or deformed nails, skin changes, fatigue, irritability, and a garlic-like breath odour due to volatile selenium metabolites; neurologic symptoms can occur in higher or sustained exposures [188].

#### 3.4.14. Strontium (Sr)

Acute exposure via dust inhalation has a relatively low acute systemic toxicity, consequently dust inhalation is mainly an irritant and nuisance dust issue [190]. Chronic exposure via dust inhalation of stable strontium is not as well characterised as many transition metals [190]. Acute exposure via hand-to-mouth transfer has only low levels of toxicity. However, chronic exposure via hand-to-mouth transfer of excess stable strontium can interfere with bone mineralisation, especially when calcium, phosphorus, or vitamin D are inadequate. The classic outcomes are strontium rickets or osteomalacia-like effects. This is most critical for children, but it can still inform risk communication for families and community exposure around dusty worksites [190,191].

#### 3.4.15. Tin (Sn)

Acute exposure via dust or fume inhalation can cause stannosis, typically described as a radiographic pneumoconiosis [192,193]. Chronic exposure via dust inhalation may also lead to stannosis, although mixed exposures can complicate outcomes [192,194]. Acute exposure via hand-to-mouth transfer mainly leads to gastrointestinal effects, with nausea, vomiting, abdominal discomfort, and diarrhoea reported after high tin concentrations in canned foods [195]. Chronic exposure via hand-to-mouth transfer typically has low systemic toxicity as inorganic tin is generally poorly absorbed; however organotin compounds are a separate hazard class with higher systemic toxicity, so speciation matters for risk interpretation [192].

#### 3.4.16. Tungsten (W)

Tungsten’s acute inhalation effects are potentiated in mixed-metal scenarios, particularly when combined with cobalt, leading to acute airway irritation and asthma-like syndromes [196,197,198]. The lung effects of chronic exposure via dust inhalation are also increased in the presence of cobalt [196,197]. Acute exposure via hand-to-mouth transfer is little studied, but with incidental ingestion more likely to be low risk than inhalation in most occupational settings, unless doses are high and bioavailable [196]. Chronic exposure via hand-to-mouth transfer is also data deficient. It is important to note that International Agency for Research on Cancer has classified weapons-grade tungsten alloy (with nickel and cobalt) as Group 2B carcinogens and cobalt metal with tungsten carbide as a Group 2A carcinogen [158].

#### 3.4.17. Zinc (Zn)

Acute exposure via dust inhalation is data deficient, but inhalation of fumes has been shown to produce “metal fume fever”, characterised by flu-like symptoms developing within hours, then usually resolving within 24–48 h [199]. Chronic exposure via dust inhalation has weaker evidence for chronic structural lung disease than for cadmium or Cr(VI), but repeated inflammatory responses are documented with inhaled zinc oxide aerosols [199]. Acute exposure via hand-to-mouth transfer at higher oral intakes can cause nausea, vomiting, and abdominal cramps, while chronic exposure via hand-to-mouth transfer can induce copper deficiency, causing anaemia, and neutropenia [200,201].

### 3.5. Toxicological Profiles of Energetic Chemicals Found in Explosive Ordnance

#### 3.5.1. 1,3-Dinitrobenzene (DNB)

Acute exposure via dust inhalation may lead to rapid-onset cyanosis and symptomatic anaemia, consistent with methaemoglobin formation and haemolysis [202]. Chronic exposure via dust inhalation is less well characterised, but repeated exposure is mechanistically expected to sustain haematologic stress (methaemoglobinemia and haemolysis) and may contribute to longer-duration reproductive toxicity signals [203]. Acute exposure via hand-to-mouth transfer may similarly produce methaemoglobinemia-related hypoxia symptoms (headache, dizziness, and weakness) and anaemia [204]. Chronic exposure via hand-to-mouth transfer may therefore drive persistent anaemia/haemolysis markers and reproductive toxicity risk where the dose is sufficient and exposure is repeated [203].

#### 3.5.2. Glyceryl Trinitrate (Nitroglycerin)

Acute exposure via inhalation (vapour/aerosol) may produce nitrate-type vasodilatory effects including throbbing headache, flushing, dizziness, and hypotension-like symptoms [205]. Chronic exposure via inhalation/dermal absorption is notable for a distinct withdrawal phenomenon resulting from rapid tolerance developed through repeated daily exposure; cessation after sustained occupational exposure can precipitate coronary vasospasm, angina, myocardial infarction, and sudden death (“Monday disease”/withdrawal ischaemia) [206]. Acute or chronic exposure via hand-to-mouth transfer (accidental ingestion) is uncommon occupationally but would be expected to produce systemic nitrate vasodilation (headache and hypotension) [207].

#### 3.5.3. Hexahydro-1,3,5-Trinitro-1,3,5-Triazine (RDX, Cyclonite)

Acute exposure via dust inhalation may lead to acute central nervous system neurotoxicity [39,40,41,42,43,44]. Chronic exposure via dust inhalation has mixed evidence, with inconsistent haematologic, hepatic, and renal abnormalities [208]. Acute exposure via hand-to-mouth transfer can cause generalised seizures, making this a high-consequence pathway in hygiene-failure scenarios [39,40,41,42,43,44]. Chronic exposure via hand-to-mouth transfer is difficult to quantify in humans, but subchronic dosing work in rats supports cumulative-dose surveillance after repeated exposure opportunities [209].

#### 3.5.4. Nitrobenzene

Acute exposure via dust or vapour/aerosol inhalation may cause clinically significant methaemoglobinemia with central cyanosis, dyspnoea, neurologic symptoms, and delayed haemolysis [210]. Chronic exposure via inhalation is associated with ongoing risk of methaemoglobinemia/haemolytic anaemia and toxic hepatitis [211]. Acute exposure via hand-to-mouth transfer may result in severe methaemoglobinemia with hypoxia symptoms (cyanosis refractory to oxygen), and recurrence can occur after large ingestions due to toxicokinetics and metabolite cycling [212]. Chronic exposure via hand-to-mouth transfer may contribute to cumulative haematologic and hepatic burden in settings where skin and surface contamination leads to secondary ingestion, aligning with repeated-exposure clinical observations and class mechanism [211].

#### 3.5.5. Nitroaromatic Toluenes (Nitrotoluenes: 2-, 3-, 4-Nitrotoluene; and 2,4- and 2,6-Dinitrotoluene [DNT])

Acute exposure via dust inhalation may lead to an oxidative “nitroaromatic” syndrome characterised by methaemoglobin formation and/or haemolysis, presenting as headache, dizziness, weakness, dyspnoea, and cyanosis-like discoloration at higher internal doses [213,214]. Comparative subchronic toxicity studies demonstrate consistent blood and spleen effects across o-, m-, and p-nitrotoluene forms, supporting a shared haematotoxic hazard profile rather than isomer-specific clinical syndromes [215,216]. For nitrotoluenes, methaemoglobin-forming potency differs by isomer in erythrocyte systems, but the clinical endpoint remains the same (functional hypoxia from methaemoglobinemia) [216,217,218]. Chronic exposure via dust inhalation can drive cumulative systemic dose with persistent haematologic and hepatic burden. For DNT specifically, worker mortality data have reported excess ischaemic heart disease in exposed cohorts, which is relevant to long-duration EOD careers where repeated low-to-moderate exposures and co-stressors (heat strain and dehydration) may amplify cardiovascular risk [219,220]. Acute exposure via hand-to-mouth transfer may produce the same methaemoglobinemia-driven toxidrome as inhalation because ingestion is an efficient route to internal dose for nitroaromatics. As such, acute symptom clusters (headache, dizziness, nausea, weakness, and cyanosis) should be treated as potentially exposure-linked in EOD contexts where eating/drinking follows dusty tasks without soap-and-water hygiene. The potential for pathophysiological effects of absorbed doses from routine handling is supported by biomonitoring work demonstrating haemoglobin adducts in workers exposed to nitrotoluenes and dinitrotoluenes, reinforcing that these chemicals can move from surface contamination to internal dose in occupational settings [221]. Chronic exposure via hand-to-mouth transfer is therefore best framed as a cumulative-dose problem with the same core targets (blood, liver, and spleen), and DNT isomer studies show that repeated dosing produces cyanosis and anaemia across isomers, underscoring that “DNT vs. nitrotoluene” is more a matter of potency and evidence depth than a fundamentally different clinical picture [222].

#### 3.5.6. Octahydro-1,3,5,7-Tetranitro-1,3,5,7-Tetrazocine (Cyclotetramethylenetetranitramine, HMX, Octogen)

Acute exposure via dust inhalation may precipitate neurotoxicity in high-intensity dust events, including seizures [124]. Chronic exposure via dust inhalation has very limited human outcome data; most evidence is extrapolated from environmental and experimental work rather than well-powered occupational cohorts [223]. Acute exposure via hand-to-mouth transfer (or accidental ingestion) is a seizure-risk pathway at sufficient dose, consistent with the human case context (heavy particulate handling) and broader toxicology signals of neurotoxicity in exposed vertebrates [124]. Chronic exposure via hand-to-mouth transfer is poorly quantified in humans, so risk should be treated as uncertainty managed and prevention focused rather than endpoint prediction confident [223].

#### 3.5.7. Pentaerythritol Tetranitrate (PETN)

Acute exposure via dust inhalation is poorly characterised in human occupational studies, but where systemic uptake occurs, PETN behaves as an organic nitrate, with acute effects including vasodilatory symptoms such as throbbing headache, flushing, dizziness, and hypotension [224,225,226,227]. Acute exposure via hand-to-mouth transfer or accidental overdose may therefore present as nitrate-type systemic effects, and organic nitrate metabolism can increase methaemoglobin [228]. Chronic occupational exposure evidence for systemic toxicity is limited; however, long-duration animal feeding studies have reported no evidence of carcinogenicity or overt toxicity at tested dietary levels, supporting the view that sustained low-level exposure may have low systemic toxicity when absorption is limited [229]. Where PETN uptake is sustained at pharmacologic doses (therapeutic context), it exerts vascular effects typical of organic nitrates, which is relevant for interpreting symptoms like headache or hypotension if unusual exposure circumstances occur [230].

#### 3.5.8. 1,3,5-Trinitrobenzene (TNB)

Acute exposure via dust inhalation may produce nitroaromatic haematotoxicity, with methaemoglobin formation, haemoglobin denaturation, and dose-dependent anaemia after short-term exposure, which may present as cyanosis–fatigue–headache after high-intensity events [231]. Acute exposure via hand-to-mouth transfer may similarly be expected to cause GI upset plus systemic hypoxia-type symptoms where methaemoglobinemia is induced (evidence base is stronger in experimental models than in well-characterised human cohorts) [231]. Chronic exposure evidence (largely animal studies) indicates persistent methaemoglobinemia and target-organ signals including kidney and spleen pathology in longer-duration studies, supporting chronic surveillance focus on haematologic indices and renal function when repeated exposure is credible [232]. Reproductive toxicity screening work has also reported methaemoglobinemia and splenic pigment changes at higher doses, reinforcing the haematologic vulnerability signal for this compound class [233].

#### 3.5.9. 2,4,6-Trinitrophenol (TNP, Picric Acid)

Acute exposure via dust inhalation may cause mucous membrane irritation (eyes and upper airway) and, if absorbed systemically at a sufficient dose, can contribute to metabolic derangement; controlled toxicokinetic work in rats demonstrated severe acidosis during acute intoxication and a relatively long plasma half-life, supporting the potential for delayed or evolving systemic presentations after a significant exposure event [234]. Acute exposure via hand-to-mouth transfer may result in gastroenteritis-like illness and systemic toxicity [235,236,237,238,239,240]. Chronic exposure via repeated skin contamination (often co-occurring with dust exposure in explosives handling) can drive dermatitis and sensitisation [241,242,243,244]. Chronic systemic toxicity evidence in humans is limited, but the combination of (i) documented acute systemic toxicity potential and (ii) established sensitisation risk supports treating repeated low-level contact as an avoidable occupational hazard requiring strict hygiene and contamination control practices.

#### 3.5.10. 2,4,6-Trinitrophenyl-N-Methylnitramine (Tetryl)

Acute exposure via dust inhalation may cause upper-airway irritation and systemic “acute illness” symptoms (headache and nausea), but the most characteristic occupational signal is acute skin and mucosal reactivity from combined dust and direct contact [245]. Chronic exposure via dust inhalation and repeated contamination events is most strongly associated with persistent or recurrent dermatitis and sensitisation-type reactions in explosives workers, sometimes at high attack rates [246]. Acute exposure via hand-to-mouth transfer may produce GI upset and systemic symptoms in high-intensity contamination scenarios, but the peer-reviewed human literature emphasises skin as the dominant clinical presentation route in occupational settings [245]. Chronic exposure via hand-to-mouth transfer is not well quantified; prevention priorities remain contamination control, glove discipline, and rapid recognition of dermatitis as an exposure sentinel event [246].

#### 3.5.11. 2,4,6-Trinitrotoluene (TNT)

Acute exposure via dust inhalation may lead to systemic nitroaromatic toxicity with methaemoglobinemia risk in high-intensity scenarios, and clinical presentation can include cyanosis, dyspnoea, and neurologic symptoms that are easy to misattribute in field settings [45,46,47,48,49,247]. Chronic exposure via dust inhalation is strongly associated with haematologic and hepatic toxicity in occupational cohorts, including toxic hepatitis and anaemia syndromes, with cataract signals also described in exposed workers [47]. Acute exposure via hand-to-mouth transfer may produce GI symptoms and systemic effects, and disposal-worker studies of nitroaromatic explosives document exposure-associated health effects consistent with systemic toxicity in occupational settings [248]. Chronic exposure assessment and biomonitoring work in TNT-exposed workers documents internal-dose markers (e.g., haemoglobin adducts and urinary metabolites) alongside recognised systemic manifestations (aplastic anaemia, toxic hepatitis, cataracts, and hepatomegaly), supporting the need for mixture-aware surveillance rather than symptom-only monitoring [249].

## 4. Exposure Pathways in Explosive Ordnance Disposal Operations

For all exposure pathways, climate triggers chiefly act by increasing mobilisation and contact. Dry, windy periods elevate dust emissions and surface loading, increasing both inhalation potential and the amount of residue available for transfer onto hands and objects [171,250]. Heavy rain, floods, and cyclones redistribute fine sediments and can remobilise contaminants, driving repeated contact with newly mobilised material during response and recovery activities [251,252,253,254]. Although direct EOD personal monitoring datasets are lacking, exposure intensity is unlikely to be uniform across tasks. The highest short-term inhalation intensity is most plausibly associated with dry excavation, brushing, grinding, cutting, and immediate post-detonation work, whereas background staging and transport contamination are more consistent with lower-intensity but repeated secondary exposure. Control use is also likely to shift exposure magnitude substantially, particularly wet suppression, plume avoidance, and respiratory protection.

### 4.1. Inhalation

Inhalation exposure in EOD work is dominated by task-driven aerosol generation plus climate-driven resuspension. Excavation, probing, shovelling, and spoil handling create mechanically generated dust that spans a wide size distribution, and the health-relevant fraction is the respirable component because it can penetrate beyond the ciliated airways into the gas-exchange region of the lung [255]. Fine particles also disproportionately carry higher concentrations of sorbed metals and corrosion products because specific surface area increases as particle size decreases [256,257]. High-energy dry tasks (grinding, demolition, and dry handling) are associated with the greatest personal dust exposures, while engineering suppression controls materially reduce airborne concentrations when consistently applied [258,259,260,261,262]. Exposure dynamics are amplified by dry season conditions, wind, and vehicle movement that increase resuspension and keep deposited dust available for repeated re-entrainment [263,264,265,266,267,268,269].

A second inhalation pathway is fume generation from hot work, including thermal cutting, grinding, and welding, all of which can volatilise metals and generate metal oxide fumes that are typically dominated by fine and ultrafine particles, thereby increasing distal airway deposition and systemic uptake potential [270,271,272,273,274]. For EOD, the practical implication is that hot work is not just a nuisance particulate hazard. It is a metal fume hazard, especially when corrosion, coatings, or mixed alloys are involved [270].

A third inhalation pathway is ultrafine particle exposure after detonation, which can generate extremely high concentrations of particles with very small diameters [275]. Ultrafine particles have distinct toxicokinetics because of their high deposition efficiency, very large surface area per unit mass, and strong inflammatory potential relative to larger particles [276,277,278,279]. For EOD operations, this supports conservative post-detonation standoff, plume avoidance, and downwind controls, particularly where soil fines and metal-bearing residues are likely entrained [275].

### 4.2. Dermal

Dermal exposure in EOD is often underestimated because gloves are assumed to “solve” skin contact. In reality, gloves create occluded microenvironments that elevate hydration, change skin pH, and can impair barrier function over repeated wear [97,280,281]. Controlled studies show that repeated glove occlusion can measurably worsen barrier integrity, including increased transepidermal water loss, and effects depend on baseline irritation and exposure duration [97,282,283]. In field conditions, occlusion is compounded by sweat, friction, and extended wear times in heat and humidity, which maps onto “wet work” style risk patterns for irritant dermatitis [282,284].

Sweat is not just moisture. It is a chemically active skin film that can increase dissolution of metal-containing particles into water-soluble ions, increasing dermal bioaccessibility [285,286]. This matters in EOD because hands repeatedly contact soils, corroded fragments, tools, and packaging surfaces. Microabrasions and minor skin damage further increase permeability. Experiments using intact versus damaged human skin show increased permeation for metal powders when the barrier is compromised [287,288]. Operationally, this supports a dermal control logic that treats gloves as one layer in a system. Fit, change-out frequency, inner liners, where appropriate, careful doffing, hand hygiene, and skin care are part of exposure control, not “comfort” measures [97,289,290,291,292,293].

Importantly, dermal contact in EOD is not only a local skin irritation issue. Some ordnance-related energetic chemicals are capable of transdermal absorption. For example, occupational biomonitoring studies in TNT- and DNT-handling workers indicate that skin uptake can be a major contributor to total absorbed dose [294,295]. Experimental studies further show percutaneous penetration of TNT, DNT, and RDX through skin models, including when residues are associated with soil, indicating that contaminated dusts and residues on skin should not be assumed to remain only on the surface [296,297,298]. Likewise, several ordnance-relevant metals and metalloids can cross skin under at least some conditions, with permeability depending strongly on chemical speciation, solubility, and barrier integrity. Reviews of human-skin studies conclude that metals are not categorically excluded by intact skin, and specific data show dermal uptake of diverse metals including arsenic, cadmium, chromium, cobalt, lead, and nickel [287,288,299,300,301,302]. For EOD practice, this means wet, sweaty, prolonged, or abrasive contact with contaminated soils, corrosion products, explosive fills, and munitions residues should be treated as a systemic exposure pathway as well as a dermatitis pathway.

### 4.3. Ocular

Ocular exposure is a pathway in EOD operations because the eye is both a directly exposed surface and a secondary contact site. Exposure can occur when dust or residue lands directly on the ocular surface during excavation, spoil handling, vehicle movement, grinding, or post-detonation plume contact. The broader ocular toxicology literature shows that particulate matter and airborne contaminants can destabilise the tear film, injure the ocular surface epithelium, and promote irritation, dry eye, conjunctivitis, blepharitis, and keratitis, with both acute irritation and longer-term inflammatory effects described across experimental and clinical studies [303,304,305,306]. Occupational reviews also note increased ocular surface vulnerability in workers exposed to dusts, fumes, and mixed chemical environments, including metal-working settings that are relevant by analogy to EOD hot work and corrosion disturbance tasks [304,307].

A second ocular pathway is hand-to-eye transfer. This matters because contaminated soil, dust, gloves, tools, radios, and vehicle touchpoints can all seed residues onto hands before transfer to the periocular region or directly to the eye. Observational studies show that adults touch their eyes repeatedly during ordinary activity, providing a clear behavioural mechanism for contaminant transfer in dirty work settings [308,309]. In EOD conditions, heat, sweat, fatigue, and discomfort are likely to increase wiping and rubbing behaviours, leading to ocular transfer during long tasks in humid environments. Although EOD-specific eye-touch frequency studies have not yet been published, this inference is consistent with the general face-touching literature [309,310].

A third pathway is transfer from contaminated PPE or from poor doffing practices. Simulation studies using fluorescent tracers have shown that removal of contaminated gloves, gowns, and other PPE frequently produces self-contamination of the face, neck, hair, and adjacent skin, supporting concern that contaminated gloves, sleeves, respirators, or brow-area PPE could secondarily contaminate the periocular area if removal sequences are poor [102,311,312]. This is directly relevant to EOD because contaminated outer gloves and face protection may be handled repeatedly during decontamination, communication, hydration breaks, and heat-management adjustments.

Sweat is also likely to act as an indirect facilitator of ocular exposure. In hot environments, sweat running from the scalp and forehead can mobilise deposited dust toward the brow, eyelid margin, and tear film while simultaneously increasing wiping behaviour that transfers contaminants from skin or glove surfaces toward the eye. This is consistent with the combined evidence for particulate ocular toxicity, frequent hand-to-eye contact, and face contamination during PPE use and removal [102,304,305,309]. Operationally, this means ocular protection should not be framed only as impact protection. It is also a contamination control measure, especially for dusty excavation, grinding, burn or blast residues, and any task where gloves or face coverings are repeatedly adjusted under heat stress [304,307].

### 4.4. Ingestion

Inadvertent ingestion is a pathway for EOD because soil and dust contaminate hands, gloves, cigarettes, drink containers, and food packaging, creating multiple hand-to-mouth and object-to-mouth opportunities [313,314]. Occupational oral exposure is shaped by behaviour, surface contamination, and hygiene controls. This aligns closely with EOD realities. Eating, drinking, use of a mobile phone, and smoking may occur in transitional spaces, including vehicles, near work zones, or during prolonged tasks when fatigue and heat drive hydration needs. Even if direct eating on task is avoided, secondary transfer can occur via hygiene failures involving contaminated handwipes, tools, vehicle interiors, radios, gloves, and shared equipment, because surface-to-hand transfer is efficient, and repeated contacts propagate contamination along touch chains [315,316,317,318,319,320,321]. Ingestion risk is most sensitive to three controls: (1) strict separation of “dirty” and “clean” zones, (2) mandatory handwashing before any hand-to-mouth activity, and (3) decontamination of tools and vehicle touchpoints to prevent recontaminating clean hands.

## 5. Acute Clinical Effects in Explosive Ordnance Disposal Personnel

Acute clinical effects in EOD personnel can arise from short, high-intensity exposures to mixed-metal particulates, energetic chemicals, and combustion products. Presentations are often syndromic rather than single agent, reflecting real-world mixtures and shared mechanisms such as oxidative stress, inflammatory activation, and mitochondrial dysfunction. However, the evidence base is uneven. Direct EOD-specific human data remain sparse. The strongest acute human evidence comes from adjacent occupational settings and case reports, particularly for metal-rich particulates, nitroaromatics, and high-dose RDX exposure, whereas some proposed syndromes remain extrapolated from animal, in vitro, or non-EOD industrial data. Acute heat strain, dehydration, sleep restriction, and exertion further complicate attribution because they can mimic or amplify toxicant-related symptoms. This section therefore distinguishes between better-established human associations and effects that are currently mechanistically plausible but not yet well demonstrated in EOD personnel.

### 5.1. Acute Cardiovascular Effects

Cardiovascular effects in EOD operations may be potentiated when task-driven dust or fume spikes that acutely perturb vascular tone and autonomic balance occur on top of heat strain, dehydration, and exertion [322,323,324,325,326]. The most convincing near-term evidence concerns inhaled particulate matter and metal-rich fumes. Human panel and cross-sectional studies in occupational groups such as welders show short-term changes in vascular function, heart rate variability, blood pressure, and endothelial biology after particulate exposure [322,323,327,328,329]. These studies support biological plausibility for acute cardiovascular strain during EOD tasks that generate dense dust or fume, but they are indirect analogues rather than EOD cohorts. By contrast, the link from brief exposure spikes to acute coronary events is supported mainly by broader particulate air-pollution epidemiology, not explosive-specific studies [322,330,331]. Energetic-related oxygen delivery failure is better established clinically. Oxidant exposures associated with nitroaromatics and related compounds can produce acquired methaemoglobinemia, creating functional anaemia and tissue hypoxia that can present as tachycardia, dyspnoea, chest tightness, hypotension, and demand ischaemia [217,332]. The suggestion that DNT contributes to ischaemic heart disease is based on observational workforce studies with inconsistent findings and likely exposure uncertainty, so it is better treated as a chronic signal than proof of a distinct acute EOD syndrome [219,333]. Likewise, severe arrhythmias from barium or arsenic reflect uncommon high-dose poisonings rather than typical field exposures [334,335,336].

These presentations can be misattributed to heat illness because the clinical phenotype overlaps substantially. Operationally, this cluster argues for a low threshold to treat chest pain, syncope, palpitations, unexplained dyspnoea, or cyanosis after high-dust, hot-work, or post-blast tasks as potential toxicant-associated events rather than just heat and to document them as exposure incidents for follow-up surveillance [322,325,326].

### 5.2. Acute Neurotoxic Syndromes

Complex exposures during fires or explosions may produce systemic symptoms and neurologic complaints alongside respiratory features [322,325,326]. Headache in EOD work is common and non-specific, but clustering can indicate an exposure driver, particularly when multiple workers in the same task cell develop headache within a narrow time window. Potential contributors include dust-associated metals, irritant gases in smoke, poor ventilation during hot work, and loud sounds. The most distinctive energetic-related neurotoxicity signal is seizure, but the human evidence is narrow. Published cases overwhelmingly involve deliberate or accidental ingestion of RDX-containing materials, often with preceding agitation, insomnia, or gastrointestinal symptoms followed by confusion and short-lived cognitive slowing [39,44,337]. Mechanistic studies showing interference with inhibitory GABAergic neurotransmission strengthen causal plausibility, but these are experimental rather than occupational field data [123]. Thus, seizure after suspected energetic exposure should be treated as a sentinel event while recognising that current evidence is strongest for high-dose ingestion and much weaker for low-level inhalational or dermal exposure during routine EOD tasks. Mixed metals can also contribute to neurotoxicity. Very high lead exposure can produce encephalopathy with altered consciousness and seizures, and acute manganese neurotoxicity is recognised but rare, again largely from non-EOD settings [338,339].

In practice, EOD surveillance should treat heat versus toxicant as a working differential until objective data are available because neither pattern is pathognomonic. This distinction matters operationally because heat stroke is immediately life threatening and requires rapid recognition. Exertional heat stroke is characterised by CNS dysfunction plus high core temperature, with multi-organ involvement that can include rhabdomyolysis, acute kidney injury, and liver injury [340,341,342,343,344,345,346]. Toxicant-driven neurotoxic events may occur without marked hyperthermia and may co-occur with prominent respiratory or oxygen delivery signals. The practical implication is that unexplained neurologic presentations in the field, especially those not tracking cleanly with heat exposure, should trigger an exposure investigation alongside urgent medical evaluation.

### 5.3. Acute Haematologic and Oxygen Delivery Effects

Nitroaromatics, including TNT, have well-described haematotoxic profiles including haemolytic and aplastic anaemia patterns, with monitoring and biomonitoring emphasised for clean-up and demolition contexts [347,348]. Evidence here is stronger than for many other acute syndromes because it includes occupational monitoring and outbreak investigations in TNT-exposed workers and military-waste disposal settings, not just mechanistic toxicology [248,347,348]. Clinically, anaemia may present as fatigue, exertional intolerance, and pallor. In the field, it can be masked by acclimatisation status and workload and becomes more conspicuous when dyspnoea or tachycardia are out of proportion to exertion. Oxidant-forming nitro compounds can also cause acquired methaemoglobinemia, producing cyanosis, headache, dizziness, dyspnoea, and low pulse oximeter readings that do not improve as expected with routine measures. The direct human evidence for this syndrome comes mainly from occupational case reports and retrospective clinical series involving nitrobenzene, aniline, and related compounds, with only limited blast-related military case literature [210,217,247,349,350]. Both inhalation and percutaneous absorption routes can be relevant for nitro compounds, and onset can follow an exposure window rather than being immediate [210,247,349,350]. For EOD, this syndrome is therefore plausible and operationally important but still extrapolated rather than directly quantified in EOD cohorts. The operational red flag is exertional dyspnoea or collapse that does not match workload, hydration status, or ambient heat index. In those cases, oxygen delivery problems should be considered alongside cardiopulmonary causes and heat illness.

### 5.4. Acute Respiratory and Mucosal Irritation

Acute inhalation of aerosols, fumes, and dusts containing mixed metals or energetic chemicals may cause upper-airway irritation, cough, wheeze, and more severe chemical injury depending on composition and dose [273,351,352]. Here, the evidence base is largely indirect, drawn from the welding fume, smoke inhalation, and particulate irritation literature rather than ordnance-specific clinical studies [273,351,352,353]. The inference is reasonable because EOD tasks can generate similar aerosol size fractions and metal-rich particles, but agent-specific dose–response data are lacking. In EOD, the highest-risk scenarios are dry excavation in hotspots and hot work on contaminated or coated surfaces. The practical implication is that respiratory symptoms clustered around cutting, grinding, or thermal work should be treated as a potential exposure event even if the task was routine. Particulate matter and combustion aerosols can also directly irritate the ocular surface, with particulate exposure leading to conjunctival irritation and inflammatory ocular surface responses, causing acute lacrimation and eye pain in dusty or smoky conditions [354]. Again, this is well supported in the broader literature but not specifically characterised in EOD cohorts. In practice, eye irritation is not just a comfort issue. It can degrade PPE adherence and increase face touching, amplifying ingestion risk.

### 5.5. Acute Hepatic and Renal Injury

Nitroaromatic energetic compounds have a long occupational history of systemic toxicity, including hepatic effects such as liver function abnormalities and liver irritation patterns [47,355,356]. Mixed-metal exposure can also contribute to hepatic stress, particularly arsenic [357]. Key nephrotoxicants in mixed dust include arsenic, cadmium, lead, and mercury, with effects ranging from acute kidney injury to chronic kidney disease, including renal failure [51,358,359,360,361,362,363,364]. Energetic chemicals such as RDX may also contribute to nephrotoxicity [365]. However, direct evidence for acute hepatic or renal injury during EOD tasks is limited. Much of the literature comes from occupational cohorts, toxicological profiles, or poisoning reports rather than event-based field studies [47,51,356,357,358,359,360,361,362,363,364,365,366]. This matters because severe heat illness, dehydration, and rhabdomyolysis can independently produce kidney and liver injury and may be more common immediate causes in the field [341,346,367,368,369,370]. For EOD, chemical nephrotoxicity or hepatotoxicity should therefore be considered contributory or co-causal unless exposure reconstruction, timing, and ancillary findings point more specifically to toxicant injury. The practical surveillance implication remains the same: acute laboratory abnormalities after field events should be interpreted alongside heat metrics, hydration status, CK, urinalysis, task logs, and any evidence of dust, fume, or residue contact.

### 5.6. Confounding, Effect Modification, and Surveillance Interpretation for Acute Effects

Interpretation of acute clinical findings in EOD settings is complicated not only by mixed exposures but also by competing causes that can produce the same symptoms, signs, or laboratory abnormalities. In the acute setting, heat illness, dehydration, sleep restriction, exertion, and infection can each present with headache, dizziness, tachycardia, cognitive slowing, fatigue, dyspnoea, renal dysfunction, and liver enzyme abnormalities. In the chronic setting, smoking, hypertension, diabetes, baseline kidney disease, chronic airway disease, prior heat illness, and non-occupational metal exposure can similarly complicate attribution of cardiovascular, renal, respiratory, and neurocognitive outcomes. These variables therefore act as confounders because they may be associated with EOD tasking while also independently contributing to the same endpoints of interest.

These same variables may also operate as effect modifiers rather than merely alternative explanations. Heat strain and dehydration are especially important because they can increase ventilation, intensify sweat-driven dermal contact, reduce renal reserve, alter toxicokinetics, and amplify the physiologic consequences of exposure. In practice, this means that the clinical significance of a given residue-contact event is unlikely to be constant across workers or across operational conditions. Susceptibility is likely to be greater in personnel with pre-existing kidney disease, hypertension, diabetes, asthma, prior exertional heat illness, or other conditions that reduce physiologic reserve. This matters because EOD exposure does not occur against a neutral physiologic background. It occurs within a susceptibility field that can magnify or obscure toxicant-related effects.

These interactions create a real risk of clinical misclassification in both directions. Toxicant-related syndromes may be mislabelled as simple heat illness, dehydration, infection, or fatigue, particularly when symptoms are non-specific and exposure reconstruction is poor. Conversely, heat-related or infectious illness may be incorrectly attributed to ordnance residues in the absence of temporal, task, or biomonitoring support. For surveillance, the practical implication is that symptoms and biomarkers should not be interpreted in isolation. Baseline assessment should capture comorbidities, prior heat illness, medications, smoking, and relevant non-occupational exposures. Incident records should capture task, weather, hydration status, PPE use, and timing of symptom onset. Abnormal findings should then be interpreted against these contextual variables, ideally with repeat testing where biomarker kinetics or evolving organ injury are plausible. This approach reduces misclassification and supports more defensible decisions about exposure attribution, fitness for duty, and escalation of controls.

## 6. Chronic Health Effects from Repeated Explosive Ordnance Disposal Exposure

Repeated EOD exposure to mixed-metal particulates, energetic chemicals, and combustion aerosols may produce chronic health effects through cumulative doses, repeated inflammatory activation, and second hits from heat strain and dehydration. In practice, chronic effects tend to track exposure drivers such as hotspot soil disturbance, dry-season dust resuspension, hot-work fumes, and post-event sediment redistribution. Outcomes are rarely single agent. They more often reflect converging mechanisms such as oxidative stress, endothelial dysfunction, mitochondrial injury, and immune dysregulation. However, the evidence base is uneven. It is strongest for metals such as lead, cadmium, arsenic, and manganese, where long-term occupational and environmental studies exist, and weaker for many energetic chemicals, where data often remain mechanistic, animal based, or derived from adjacent munitions industries rather than EOD personnel themselves. Chronic outcomes are also difficult to attribute because cardiovascular, renal, and neurocognitive endpoints are influenced by age, smoking, heat, dehydration, comorbidity, and co-exposures. This section therefore prioritises associations with the most consistent human evidence while identifying pathways that are currently more inferential. For chronic outcomes, these same factors operate not only as confounders but also as modifiers of susceptibility, meaning that surveillance and attribution require repeated contextual data rather than endpoint interpretation in isolation.

### 6.1. Chronic Cardiovascular Effects

In chronically exposed EOD personnel, cardiovascular morbidity may be driven by a combined burden of metal uptake and repeated inhalation of metal-rich particulate generated during excavation, brushing, post-blast inspection, and resuspension from vehicles and staging areas. Across the metals most encountered in EOD contexts, lead has the clearest and most consistent cardiovascular evidence, supported by systematic review-level human data linking exposure with hypertension and vascular dysfunction [176]. Cadmium also has a growing human evidence base, including systematic reviews and dose–response meta-analysis, although heterogeneity in exposure metrics and confounder control remains [371,372]. Arsenic has long been associated with peripheral vascular disease and broader atherosclerotic outcomes, particularly at higher chronic exposure levels than many modern occupational settings [373]. For EOD, these metal-related effects are plausibly amplified by inhalation of fine and ultrafine particulate, but the most directly relevant occupational analogue remains the welding fume literature rather than EOD-specific cohorts [322,374]. Mixture-oriented epidemiology further supports the idea that metals act jointly, but most such studies come from general population datasets, so translation to tactical occupational exposure patterns should be cautious [375,376]. By contrast, direct chronic cardiovascular toxicity evidence for classic energetic chemicals remains limited. Perchlorate-related thyroid disruption is mechanistically clear, but its downstream cardiometabolic significance in EOD workers is more indirect than demonstrated [377,378]. Clinically, surveillance should therefore focus on cumulative cardiovascular risk markers while avoiding overstatement that a single exposure signature has been proven for EOD.

### 6.2. Chronic Renal Trajectories

Kidney risk may be central for mixed-metal exposure because several common metals concentrate in renal tissue and can have long biological half-lives, so body burden can accumulate across years of repeated exposure [379,380,381,382,383]. Among chronic outcomes, renal toxicity has one of the stronger mechanistic and epidemiologic bases for mixed-metal exposure. Lead has been linked to lower kidney function and increased chronic kidney disease risk across occupational and population studies, although susceptibility appears greater in workers with pre-existing hypertension, diabetes, or reduced renal reserve [50,51,379,380,384,385]. Cadmium has especially strong evidence for tubular toxicity and albumin handling disruption, with biomarker-based monitoring long embedded in occupational practice [381,382,383,386]. What is not yet known is whether EOD exposure patterns are sufficient to reproduce these trajectories at meaningful rates, because longitudinal EOD cohorts are lacking. In EOD, the practical concern is that repeated moderate exposure episodes could, over years, shift workers toward lower renal reserve, with dehydration and heat stress then producing disproportionate functional drops and kidney injury risk [387,388,389]. Nephrotoxicity biomarkers including urinary β2-microglobulin, the albumin-to-creatinine ratio, and related markers of tubular handling are useful for early detection [382,390]. Interpretation should remain explicitly contextualised to both exposure and thermal strain, including blood lead trends and urinary cadmium as a proxy for body burden [51,391].

### 6.3. Chronic Neurocognitive Outcomes

Chronic metal exposure is associated with neurocognitive shifts, including decrements in attention, processing speed, working memory, cognitive control, and reaction time, all of which may influence occupational performance in ways that increase error probability under time pressure [392,393,394,395,396,397,398,399,400]. The most robust occupational evidence comes from manganese-exposed workers, especially welders, where meta-analyses support small-to-moderate decrements across several cognitive domains [393,395,396,397,398,399,400]. These findings are relevant to EOD because they involve aerosol-generating metal work, but they still derive from adjacent occupations rather than EOD cohorts. Effect sizes are generally subtle and often subclinical, so translating them into fitness-for-duty decisions requires care [398,399,400]. The evidence for chronic neurocognitive effects from energetic chemicals at typical occupational exposure levels is far thinner. Even so, in safety-critical work, modest cognitive change may matter operationally because higher cognitive failure rates are associated with higher odds of injury and near-miss events [394,401,402]. This supports adding neurocognitive endpoints to surveillance in higher-exposure roles but not a claim that chronic neurocognitive decline has already been demonstrated specifically in EOD personnel [403,404]. Fitness for duty should therefore be framed as capability and risk tolerance anchored to job demands and hazard context, with exposure reduction and reassessment preferred over simplistic worker removal when subtle changes are detected [403,404,405,406,407].

### 6.4. Chronic Haematologic and Immune Effects

Both mixed-metal and energetic-chemical occupational exposures can lead to haematological or immunological outcomes, but the strength of evidence differs by endpoint. Chronic nitroaromatic-related haematotoxicity is supported by occupational studies in TNT-exposed and military-waste workers, making anaemia one of the better-substantiated non-neoplastic outcomes for energetic chemicals [46,248,347,356,408]. In EOD, chronic anaemia risk is most plausible when repeated contact occurs and dermal and ingestion pathways are not tightly controlled [248,313,408]. By contrast, the immune evidence for lead is more heterogeneous and is based largely on cytokines and inflammatory biomarkers rather than hard clinical immune disease endpoints [409,410,411]. Clinically, persistent anaemia can degrade endurance, reduce physical work capacity, and worsen heat tolerance during sustained field operations by shrinking aerobic reserve in conditions that already elevate cardiovascular strain [91,412,413,414]. The immunological relevance is therefore less about diagnosing a discrete immune disease and more about recognising that low-grade inflammation may amplify fatigue, impair recovery, and interact with heat strain [415,416]. Persistent fatigue remains non-specific and should trigger review of exposure controls, sleep and heat management, and medical assessment for anaemia and renal function rather than being interpreted as toxicant related by default.

### 6.5. Chronic Endocrine Disruption

Endocrine disruption is biologically plausible for both metals and energetic chemicals, but much of the evidence remains observational or experimental rather than EOD specific. Associations between lead exposure and altered semen parameters are supported by systematic review-level human data, although individual studies vary in exposure assessment and adjustment for confounders [417]. Cadmium has substantial experimental and epidemiologic literature on steroidogenesis and reproductive effects, but causal translation to real-world occupational fertility risk is less certain [418,419,420,421]. Among energetic-related agents, perchlorate has the clearest mechanism through inhibition of thyroid iodide uptake, and recent human systematic review evidence supports an association with thyroid function markers, especially in susceptible populations [129,378,422,423,424,425]. Even so, this remains an indirect pathway for most EOD programs unless water or other repeated exposure routes are documented. For EOD programs, targeted monitoring is most justified where repeated environmental exposure pathways exist and where vulnerable groups, particularly pregnancy, may be present in the same operational footprint [424,425].

### 6.6. Chronic Cancer Risks

Long-latency cancer risk is difficult to quantify in EOD because exposures are variable, mixtures are complex, latency is long, and historical monitoring is often limited. The strongest evidence in this section therefore comes not from EOD cohorts but from authoritative carcinogenic classifications. Several metals relevant to mixed-metal dust are established or probable human carcinogens, including arsenic, cadmium, chromium (VI), inorganic lead compounds, and nickel compounds [178,426,427]. More recent IARC evaluations also highlight concern for cobalt and antimony compounds relevant to some munitions profiles [158,426]. Direct military or ammunition worker evidence is much thinner. The recent report of increased bladder cancer incidence among British Army ammunition technicians is important because it is one of the few published occupation-specific signals, but it should currently be treated as trade-group specific rather than definitive for EOD more broadly [428].

EOD personnel health monitoring should therefore be built around longitudinal monitoring and exposure registries, not because every chronic association is already proven in EOD but because the combination of plausible hazard, limited historical exposure data, and long disease latency makes retrospective reconstruction otherwise difficult [429,430,431,432,433]. A registry supports exposure reconstruction, early detection, and compensation pathways if long-latency disease emerges. It also enables continuous improvement by linking health endpoints back to task conditions, climate triggers, and control performance. In EOD, that is a precautionary occupational health measure rather than optional research.

## 7. Translational Surveillance and Control Priorities for EOD Programs

**Figure 1:**
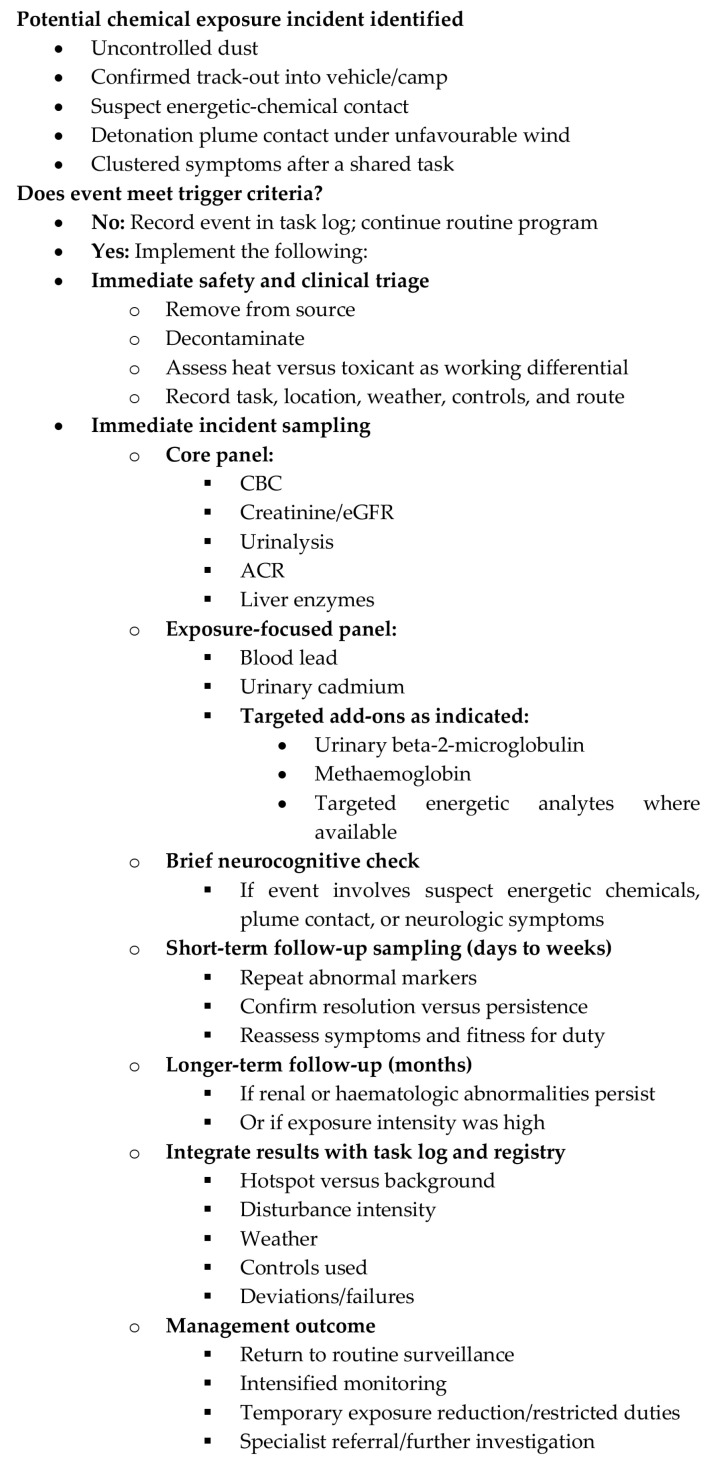
Generic incident response flowchart for high-residue events in explosive ordnance disposal operations.

Taken together, the evidence reviewed above supports a practical occupational health response built around three linked elements: risk-based surveillance, targeted biomonitoring, and control measures directed at the exposure pathways most likely to drive internal dose in EOD work. Surveillance should not be generic or calendar-based alone. Instead, it should be linked to task type, hotspot status, operational tempo, seasonal conditions, and incident history. A tiered approach is therefore the most defensible model, with baseline assessment for all personnel, more frequent periodic surveillance for higher-exposure roles, and incident-triggered escalation after uncontrolled dust events, suspect energetic-chemical contact, or detonation plume exposure. This is especially important because the same clinical syndromes may reflect mixed-metal or energetic-chemical exposure, heat strain, dehydration, or combinations of these stressors, making context-linked interpretation essential [51,176,341,382]. A task-specific flowchart should be designed (generic version shown in Figure 1).

Biomonitoring should remain focused on markers that are both informative and operationally interpretable. A practical core program includes full blood count, creatinine with estimated glomerular filtration rate, urinalysis, urine albumin-to-creatinine ratio, liver enzymes, blood lead, and urinary cadmium. Within this set, blood lead is a high-utility marker for exposure trend assessment, while urinary cadmium is more informative for longer-term body burden and tubular risk in relevant exposure contexts [51,382,391]. Targeted markers such as urinary beta-2-microglobulin, methaemoglobin, and selected energetic-related analytes are best reserved for higher-risk deployments, symptom clusters, or post-incident investigation rather than routine universal testing. The key principle is that biomarkers should not be interpreted in isolation. Their value depends on linkage to baseline values, symptom inventories, task logs, environmental conditions, and known control failures.

Priority controls should focus on the routes most likely to sustain cumulative or incident-related uptake: inhalation of dusts and metal fumes, dermal loading under hot and occlusive glove conditions, and ingestion through contamination transfer. The highest-priority measures are therefore strict dirty–clean zoning, decontamination at hotspot boundaries, wet suppression where safe, enforced hand hygiene before eating or drinking, respiratory protection matched to task, glove systems designed for both protection and safe doffing, and measures that prevent secondary transfer into vehicles, camps, and homes [68,82,102,317,434,435,436]. These controls should not be framed as optional hygiene advice. They are part of operational capability. Their effectiveness depends on consistent implementation, auditing, and feedback from surveillance data.

The central translational message is integration. Surveillance should inform control refinement, and control failures should trigger targeted follow-up assessment. In this way, EOD programs can move beyond passive detection of illness and toward a more preventive model in which exposure signals, early biological change, and operational controls are interpreted together. For a workforce operating in safety-critical conditions, this integrated approach offers the most defensible framework for protecting health, preserving readiness, and improving long-term occupational risk management.

## 8. Biomonitoring and Medical Surveillance Design

A biomonitoring and medical surveillance program for EOD personnel should be built around the reality of mixed exposures (metals, conditional energetics, and combustion aerosols) and the operational drivers that modulate dose (dry-season dust, hot work, post-event redeployment, and hygiene constraints) (Table 1). The design goal is not “diagnosis by spreadsheet”. It is early detection of meaningful change, defensible linkage to task-based exposure, and a feedback loop that strengthens controls. A tiered model is most efficient. It assigns more intensive monitoring to higher-exposure roles, hotspot deployments, and post-incident conditions.

### 8.1. Baseline Assessment

#### 8.1.1. Exposure History

Establish a structured baseline exposure profile rather than a narrative. Capture years in EOD, typical task mix (excavation, hot work, demolition support, and post-event response), frequency of hotspot work, and past known incidents (suspect residues, uncontrolled dust, and detonation plume contact). Include non-work exposures that may confound interpretation, including hunting as a lead source, welding or battery work, and household water composition. This is essential because biomarkers such as blood lead reflect multiple inputs and are most interpretable when baseline sources are understood [176].

#### 8.1.2. Medical History and Comorbidities

Focus on conditions that alter susceptibility or interpretation. Kidney disease, hypertension, diabetes, and recurrent dehydration are major modifiers of renal vulnerability. Asthma and chronic rhinitis increase airway symptom burden and can mimic irritant injury. Prior heat illness episodes are critical because repeated exertional heat stress can influence renal trajectories and confound symptom interpretation in the field [341]. Document medication use that affects kidney function or oxidative stress handling.

#### 8.1.3. Baseline Laboratory Panels

Baseline testing should be practical and aligned to likely endpoints. Minimum baseline panels typically include full blood count, creatinine with estimated glomerular filtration rate (eGFR), urinalysis and urine albumin-to-creatinine ratio (ACR), and liver enzymes. Metals biomonitoring should be targeted rather than exhaustive. Blood lead is a high-utility marker, while urinary cadmium can be used to reflect longer-term body burden and tubular risk in relevant contexts [437]. Establish a baseline symptom inventory and a brief cognitive screen (reaction time and attention) to support later change detection.

### 8.2. Periodic Surveillance

#### 8.2.1. Frequency Based on Operational Tempo

Monitoring frequency should be tied to exposure intensity. A workable rule set is: annual surveillance for low-exposure roles, six-monthly for routine field roles with periodic hotspot work, and quarterly or campaign-based surveillance for teams doing repeated hotspot clearance in dry-season conditions or frequent post-event deployments. This risk-based approach aligns with occupational monitoring principles that emphasise targeting higher-risk cohorts and integrating exposure context into medical interpretation [438].

#### 8.2.2. Symptom Inventories

Use short, repeatable tools that map to operationally relevant domains: respiratory irritation and wheeze, headache clusters, skin symptoms under gloves, sleep disruption, fatigue, and neurocognitive complaints (attention slips and slowed decisions). Cluster detection is important. Multiple workers with the same symptom pattern after a shared task is an exposure signal, even when individual symptoms are mild.

#### 8.2.3. Tiered Laboratory Testing

A tiered model improves efficiency. Tier 1 is a standard panel for all monitored workers (CBC, creatinine/eGFR, urinalysis and ACR, and liver enzymes). Tier 2 is activated by high-risk deployments or symptom clusters and adds exposure-focused measures such as blood lead, urinary cadmium, and, when justified, targeted tubular markers (for example, β2-microglobulin). Tier 3 is incident driven and includes more intensive testing (see Section 8.3). The goal is to detect early renal or haematologic change before it becomes performance limiting.

### 8.3. Post-Incident Protocol

#### 8.3.1. Triggered Sampling After High-Residue Events

Define “incident” broadly enough to capture chemical exposure. This includes uncontrolled dust during hotspot work, confirmed track-out of contaminated dust into vehicles or camp, suspect energetic-chemical contact, and close proximity to detonation plume under unfavourable wind. Sampling should occur as soon as feasible, with a second sample at a defined follow-up window because some biomarkers reflect different kinetics (fast redistribution versus slower integration).

#### 8.3.2. Neurocognitive Check

After any event involving suspect energetic chemicals, detonation plume contact, or neurotoxicity symptoms, conduct a brief cognitive screen. This can be as simple as a reaction time and attention task plus symptom checklist. The point is not diagnosis. It is documenting change relative to baseline and ensuring safe return to duty.

#### 8.3.3. Follow-Up Intervals

Follow-up should be staged: immediate assessment, a short-term follow-up (days to weeks) to confirm resolution or trend, and a longer follow-up (months) where renal or haematologic signals were abnormal. For heat-associated incidents, follow-up should explicitly consider combined injury pathways because exertional heat stress can cause multi-organ effects that may evolve after the event [439,440,441,442].

### 8.4. Data Integration

#### 8.4.1. Linking Task Logs to Clinical Results

Biomonitoring has limited value if it is not integrated with exposure drivers. Each clinical record should be linkable to task logs that include location context (hotspot vs. background), disturbance intensity, weather, controls used (wet methods and decontamination), and any deviations. This enables exposure reconstruction and defensible interpretation.

#### 8.4.2. Exposure Registry Model

Build a registry that holds de-identified analytics and a protected clinical layer. The analytics layer supports trend plots by task category, season, and hotspot corridor and drives exposure management plan improvement. The clinical layer supports individual care and fitness-for-duty decisions.

#### 8.4.3. Confidentiality Considerations

Confidentiality must be explicit. Workers should understand who can see what, and how results are used. Registry governance should align with occupational health ethics: minimum necessary access, clear consent, and use of aggregated reporting for management decisions to avoid stigma while still improving controls.

## 9. Prevention and Control Strategies in Explosive Ordnance Disposal Settings

Prevention in EOD settings needs to be built around the exposure realities that drive internal dose. These are disturbance of hotspot soils, dust resuspension during dry and windy periods, hot-work fumes, post-detonation fine and ultrafine aerosols, and hand-to-mouth transfer under constrained hygiene. Controls should follow the mitigation hierarchy and be implemented as a system, not as isolated measures. The most defensible approach is to treat chemical exposure controls as an operational capability. They should be planned, resourced, audited, and linked to surveillance outcomes. Overall, prevention works when controls are integrated into the exposure management plan as enforceable work processes, not optional add-ons. Continuous improvement should be driven by monitoring results, incident reviews, and worker feedback [436,443].

### 9.1. Engineering Controls

#### 9.1.1. Wet Methods, Where Safe

Wet suppression is the dominant engineering control for dust because it reduces aerosol generation at source and interrupts resuspension. The constraint in EOD is compatibility with energetic safety. Wetting must be applied where it does not increase hazard, such as on spoil piles, access tracks, and decontamination zones, and must not be used to disturb suspect residues unless authorised. Where wetting is feasible, use low-pressure spray or misting to avoid dispersal, and maintain a moisture threshold rather than saturating soils. When wetting cannot be used, consider alternative physical suppression methods such as temporary covers, soil stabilisation products suitable for the setting, or controlled access to limit traffic [444,445].

#### 9.1.2. Decontamination Stations

Decontamination is essential because it breaks the transfer chain from hotspot soil to hands, tools, vehicles, and living areas. At minimum, establish staged decontamination points at the boundary of hotspot zones, with clear sequencing for tool wash-down, boot cleaning, and doffing of outer layers. Include sealed waste containers for contaminated wipes and disposable PPE. For vehicle operations, include wheel and undercarriage controls where track-out risk is high. The key design principle is that decontamination steps must be easier to follow than to bypass. This approach is consistent with evidence that contaminated PPE removal commonly causes self-contamination and that contaminated dust can be transferred into vehicles and onward to households unless decontamination barriers are effective [102,434,435].

#### 9.1.3. Dirty–Clean Zoning

Zoning is a core exposure control because it reduces cross-contamination and ingestion risk. Implement physical delineation of dirty zones, transition zones, and clean zones. Use signage, ground marking, and dedicated storage. Keep eating and smoking only in clean zones. This is not just hygiene: it is exposure prevention by design. The rationale is supported by evidence on inadvertent occupational ingestion via contaminated hands and objects and by the broader take-home exposure literature showing that contaminants are redistributed when spatial barriers between contaminated and clean activities are weak [317,435,446].

### 9.2. Administrative Controls

#### 9.2.1. Hygiene Enforcement

Administrative controls fail when they are written but not operationalised. Hygiene needs checklists, supervision, and audit. Establish mandatory handwashing or hand decontamination before any hand-to-mouth activity. Include explicit no-exceptions rules for eating, drinking, vaping, use of mobile phones, or smoking outside clean zones. Build in time and water availability so hygiene is achievable under heat stress. If hygiene is not feasible due to water limits, treat that as an operational stop condition, not a workaround. This is supported by occupational studies showing that adult workers do exhibit measurable hand-to-mouth and object-to-mouth behaviour and by reviews emphasising that hand hygiene is important but cannot substitute for sound work practices and contamination control [317,446,447].

#### 9.2.2. Task Rotation

Rotation reduces the cumulative dose when peak exposures are task specific. It is most effective for dust-intensive work, hot work, and post-event sediment handling. Rotation only works if it is paired with exposure-aware scheduling. Moving workers between two high-exposure tasks does not reduce dose. The practical model is to alternate high-exposure tasks with low-exposure tasks, then verify with personal monitoring and symptom logs. More broadly, the intervention literature supports planned, monitored workplace interventions, while the job-rotation literature shows that rotation reduces exposure only when schedules are designed around differential hazard intensity rather than simple personnel swapping [436,448,449].

#### 9.2.3. Eating and Smoking Separation

Separation must be physical and procedural. Provide clean hydration and eating spaces with closed storage, dedicated utensils, and no contaminated gear. Implement a rule that gloves and tools do not enter the eating area. This reduces ingestion risk and reduces cross-contamination to personal items. The logic is supported by occupational hand-to-mouth behaviour data and by take-home exposure studies showing that contamination transfer occurs across settings unless clean-use boundaries are actively maintained [317,435,446].

### 9.3. Personal Protective Equipment Optimisation

#### 9.3.1. Glove System Selection

Gloves should be treated as a system. Consider inner liners to reduce sweat-driven irritation and improve doffing, plus an outer glove chosen for abrasion resistance and task dexterity. Set change-out rules based on time, contamination, and glove damage. Doffing technique is critical because self-contamination often occurs during glove removal. Provide training and spot checks. The importance of glove removal technique is strongly supported by simulation studies showing frequent contamination of hands and forearms during PPE removal, with substantial improvement after training interventions [102,311].

#### 9.3.2. Respiratory Protection by Task

Respiratory protection needs to be matched to the aerosol type. Dust-generating excavation requires filtration aligned to respirable particles. Hot work can add metal fumes and ultrafine particles, increasing the need for higher-performance filtration and better face sealing. Where detonation aerosols are relevant, plume avoidance is the primary control, with respiratory protection as a secondary control in the residual-risk space. Fit testing, seal checks, and heat-tolerant wear strategies matter because non-compliance is often driven by discomfort and thermal burden rather than intent. The relevance of ultrafine particles in thermal metal work is well established in welding studies, and respirator-wear burden has also been demonstrated in occupational studies of prolonged respiratory protection use [68,450,451,452].

#### 9.3.3. Change-Out and Cleaning Protocols

Filters, reusable respirators, and eye protection need defined change-out and cleaning cycles. Establish dirty storage bags for used PPE and clean storage for serviced PPE. Make cleaning supplies available at decontamination points and in camps. Without this, PPE becomes a contamination vector rather than a control. This is consistent with the PPE-doffing literature and with intervention reviews showing that PPE effectiveness depends heavily on maintenance, training, and implementation fidelity rather than simple provision of equipment [102,436].

### 9.4. Secondary Exposure Prevention

#### 9.4.1. Vehicle Contamination Control

Vehicles are major transfer amplifiers because they spread contaminants across workers and time. Use washable floor mats, dedicated dirty-gear storage, and routine wet cleaning (never compressed air cleaning). Keep drinking water and food out of vehicles used in hotspot work. If vehicles must be used for mixed purposes, enforce cleaning gates before clean use. This is directly supported by studies showing substantial lead dust contamination in workers’ vehicles, with clear implications for ongoing occupational and para-occupational transfer [434,435].

#### 9.4.2. Laundry Management

Clothing can transport dust to sleeping areas and to households. Establish onsite laundry or bag-and-transfer protocols that keep contaminated clothing separate. Avoid shaking dry clothes. Use wet handling. Provide dedicated work clothing and ensure adequate spares so workers are not forced to re-wear contaminated gear. This is well aligned with the take-home exposure literature, including lead case investigations showing contamination moving from workplace to home environment and affecting family members [82,435].

#### 9.4.3. Home Exposure Minimisation

For local staff, provide simple guidance and resources to prevent take-home exposure: changing and washing at the site when possible, bagging work clothes, and keeping boots and tools outside living spaces. This is ethically important because it reduces secondary exposure to families and strengthens community trust. The peer-reviewed literature on take-home exposure strongly supports this emphasis, particularly for metals and dusts that can persist on clothing, vehicles, and domestic surfaces [82,434,435].

## 10. Evidence Gaps and Research Agenda

### 10.1. Data Deficient Areas

The scientific evidence base for mixed-metal and energetic-chemical hazards in EOD work is adequate to justify control, surveillance, and precautionary decision making, but it remains incomplete in the areas that matter most for defensible risk characterisation. The dominant gaps are not “what is toxic”. They are uncertainty in task-specific doses, the health relevance of real-world mixtures, and the effectiveness of controls under tropical field constraints. A structured research agenda should therefore focus on prospective cohorts, mixture-focused biomarkers, task-based monitoring, and field-validated interventions and detection tools. The research agenda should be framed as an implementation science program: quantify task exposures and dose, validate mixture-sensitive biomarkers, and test controls and field tools in the exact operational contexts where exposure is most likely.

### 10.2. Need for Prospective EOD Cohorts

Much of the available evidence comes from case reports, analogous occupations (construction dust, welding fumes, and ammunition manufacture), or environmental studies. EOD-specific longitudinal cohorts are rare, limiting inference about exposure–response relationships, thresholds for subclinical change, and cumulative risk trajectories. Prospective cohorts that follow EOD personnel across seasons and deployment cycles can quantify within-person change in renal function, haematologic indices, and neurocognitive performance and can identify modifiers such as heat strain and dehydration. Cohort designs are also essential for separating exposure effects from baseline risk factors and for building defensible fitness-for-duty thresholds.

### 10.3. Mixture-Specific Biomarker Development

Current biomonitoring is dominated by single analytes (blood lead and urinary cadmium) and generic clinical panels (CBC and creatinine). This is insufficient for explosive ordnance mixtures where multiple metals and energetic chemicals converge on shared mechanisms such as oxidative stress, mitochondrial dysfunction, and endothelial injury. There is a need for biomarkers that better reflect mixture burden and early effect, including panels that capture tubular stress, oxidative damage, and inflammatory activation. “Exposure plus early-effect” biomarker sets could be developed and validated against task-based exposure metrics to improve sensitivity for subclinical change and to guide control escalation.

### 10.4. Task-Based Exposure Monitoring

Exposure assessment in EOD needs to move beyond general site characterisation and toward task-resolved personal monitoring. Priority measures include personal inhalable and respirable dust monitoring with metals on filter for hotspot disturbance tasks, as well as combined particulate and vapour phase monitoring for energetic-chemical exposures, wipe sampling for transfer pathways (hands, tools, and vehicle touchpoints), and targeted event-triggered sampling after cyclones, floods, or detonation activities. Monitoring should be integrated with structured task logs and weather data to support quantitative models of likelihood and to inform stop-work thresholds.

### 10.5. Intervention Efficacy Trials

Engineering and administrative controls are widely recommended, but evidence of their real-world effectiveness in tropical EOD conditions is thin. Controlled field trials should evaluate wet methods and dust suppression approaches, hygiene and decon system designs, glove system configurations, and respiratory protection strategies under heat and humidity. Outcomes should include reductions in personal exposure metrics and improvements in biomonitoring trends, not just compliance.

### 10.6. Field-Appropriate Rapid Detection Technologies

Rapid screening for metals and energetic chemicals can transform decision making after trigger events and during hotspot work. Research should prioritise ruggedised methods suitable for remote settings: portable X-ray fluorescence (XRF) for metals, rapid immunoassays or electrochemical sensors for nitroaromatics, and simplified sample workflows that link to confirmatory laboratory analysis. The key requirement is not maximum sensitivity. It is operational reliability, clear decision thresholds, and integration into exposure management plan governance.

## 11. Conclusions

Mixed-metal and energetic-chemical exposure in EOD operations is measurable and modifiable. EOD work routinely places personnel in contact with mixed-metal residues, dust-associated contaminants mobilised by disturbance, and, in conditional contexts, energetic chemicals and combustion by-products. In tropical environments, these hazards are amplified by heat, humidity, heavy rainfall, and cyclone-driven redistribution that increase both mobilisation and contact pathways. The implication is straightforward. Mixed-metal and energetic-chemical exposure risk in EOD is not an abstract environmental issue. It is an operational exposure problem that can be managed with the same discipline applied to explosive safety.

A mixture-aware approach improves both clinical safety and incident interpretation. Field presentations are often syndromic. Headache clusters, irritant respiratory symptoms, unexpected fatigue, neurotoxic events, or oxygen delivery abnormalities may reflect combined exposures rather than a single agent. Mixture-aware clinical reasoning helps distinguish toxicant-driven syndromes from heat illness and supports earlier escalation when patterns are inconsistent with simple exertional stress. It also strengthens exposure reconstruction, which is essential for defensible decisions about stop-work triggers, redeployment after extreme weather, and corrective actions under the Environmental Management Plan.

Surveillance and hygiene protocols are the practical bridge between risk characterisation and harm reduction. A tiered medical surveillance program, tied to operational tempo and hotspot work, can detect early renal, haematologic, and neurocognitive changes before they become performance limiting. Biomonitoring provides an objective complement to symptoms but only when linked to task logs, weather conditions, and control performance. Hygiene systems and decontamination are equally central because they interrupt the transfer chains that drive ingestion and secondary exposure through tools, vehicles, and living areas. When clean–dirty zoning, wet methods where safe, and verification monitoring are consistently applied, residual risk can be driven down substantially.

The broader call is for routine integration of occupational hygiene and toxicology into EOD programming. Mixed-metal and energetic-chemical risk should be managed as a core capability, not an optional add-on. That means receptor-specific risk matrices that inform task planning, exposure monitoring that is task resolved and event triggered, and a governance framework that links surveillance findings back to control improvements. Embedding these elements strengthens worker protection, improves operational continuity, and builds community trust in EOD outcomes.

## Figures and Tables

**Table 1:** Biomonitoring framework for explosive ordnance disposal personnel.

Biomarker/Test	Matrix	Primary Purpose	Best Use in Program	Key Interpretation Note
Blood lead	Whole blood	High-utility marker of lead exposure and exposure trend	Baseline, periodic surveillance, and post-high-dust/fragment/plume events	Most informative when interpreted against personal baseline, task history, and non-occupational lead sources
Urinary cadmium	Urine	Reflects longer-term body burden and tubular risk	Baseline in relevant roles, periodic surveillance, and renal-focused follow-up	Better for cumulative exposure than for immediate short-term spikes
Creatinine and eGFR	Blood	Renal function screening	Baseline for all personnel, periodic surveillance, and post-incident follow-up	Must be interpreted alongside hydration status, heat strain, and exertion
Urinalysis	Urine	Broad kidney and urinary tract screening	Baseline, routine periodic surveillance, and incident follow-up	Useful as a simple first-line screen but non-specific alone
Urine albumin-to-creatinine ratio (ACR)	Urine	Early glomerular renal injury signal	Baseline, periodic surveillance, and post-incident renal follow-up	Best interpreted as a trend rather than a single isolated value
Urinary beta-2-microglobulin	Urine	Early tubular injury marker	Tier 2 or Tier 3 testing where hotspot exposure, repeated dehydration, or cadmium concern is credible	Most useful as a targeted renal marker rather than a universal routine test
Full blood count (CBC/FBC)	Blood	Screens for anaemia and other haematologic effects	Baseline for all personnel, periodic surveillance, and incident follow-up where nitroaromatic exposure is plausible	Particularly relevant where TNT or related nitroaromatic exposure is suspected
Liver enzymes	Blood	Screens for hepatic stress or injury	Baseline, periodic surveillance, and post-incident follow-up	Should be interpreted with heat illness, rhabdomyolysis, and exposure reconstruction in mind
Methaemoglobin, ideally by co-oximetry	Blood	Detects oxygen delivery impairment after nitroaromatic-type exposure	Incident-driven only, especially with cyanosis, unexplained dyspnoea, low SpO2, or suspected energetic/nitroaromatic contact	Not recommended as a routine surveillance marker
Targeted urinary energetic metabolites where analytically available	Urine	Supports exposure confirmation for selected energetic compounds	Incident-driven or research-intensive programs	Best framed as confirmatory or special purpose rather than core routine surveillance

## Data Availability

No new data were created or analyzed in this study. Data sharing is not applicable to this article.
